# Editorial: Carbapenem-resistant *Enterobacteriaceae* in the Asia Pacific and beyond, volume II

**DOI:** 10.3389/fmicb.2022.1115627

**Published:** 2023-01-04

**Authors:** Yi-Wei Tang

**Affiliations:** Medical Affairs, Danaher Diagnostic Platform/Cepheid, Shanghai, China

**Keywords:** carbapenem-resistant *Enterobacteriaceae*, Asia Pacific area, carbapenemase, *K. pneumoniae*, *E. coli*

*Enterobacteriaceae* are the cause of both community-acquired and hospital-acquired infections. These infections can range from minor to life-threatening disorders, especially among vulnerable populations, including infants, elderly persons and patients with immunodeficiency. β-lactam drugs are often the primary therapeutic option for serious *Enterobacteriaceae* infections, and the carbapenems are usually considered as the last resort antibiotics against complicated infections. Unfortunately, carbapenem-resistant *Enterobacteriaceae* (CRE) has now emerged worldwide as an urgent antibiotic resistance threat. Asia-Pacific countries are regarded as high CRE prevalent regions, and the rate of CRE detection has been continuously increasing. Unlike some other regions in the world, e.g., the United States, where single class KPC carbapenemase is predominant, different classes of carbapenemase (e.g., KPC, NDM, IMP, VIM, and OXA48) are frequently identified in regions within Asia-Pacific. CRE are particularly concerning, as the resistance is encoded by plasmid-borne genes, and can disseminate clonally or horizontally. A number of plasmid-mediated or clonal CRE outbreaks have been documented in several Asia-Pacific countries, such as China. In addition, the plasmid-mediated colistin resistance gene, mcr-1, which was originally identified in China, has been found in CRE clinical isolates, posing another significant threat to global health. Moreover, hypervirulent *K. pneumoniae* strains, associated with life-threatening community-acquired infections in young and healthy hosts, have been frequently identified in Asia-Pacific region. More recently, hypervirulence has emerged in carbapenem-resistant *K. pneumoniae* strains in China, representing a “triple threat” (hypervirulent, multidrug resistant, and highly transmissible) to global public health. CRE studies on resistance mechanisms, epidemiology, diagnosis and treatment in the Asia-Pacific region are making significant progress, and now is the time to arrange a Research Topic to present and highlight these novel findings.

Due to the success of the previously successful Research Topic on *Carbapenem-Resistant Enterobacteriaceae in the Asia Pacific and Beyond* we have launched a volume II of the collection. The present Research Topic of Frontiers in Microbiology entitled *Carbapenem-resistant Enterobacteriaceae in the Asia Pacific and beyond, volume II* featured eight authoritative articles on etiology, pathogenesis, virulence, resistance, diagnosis, treatment and control of CRE in the Asia-Pacific region. Schäfer et al. from the Hannover Medical School of Germany examined physiological adaptation of *Escherichia coli* clinical isolates exhibiting two distinct resistance mechanisms, either carrying a carbapenemase or alterations of porin-encoding genes, during growth with sublethal concentrations of ertapenem in chemostat culture. The authors demonstrated that strategies to deal with the antibiotic were distinct between strains of the two groups, where expression of carbapenemases was the only response in CARB, whereas wide-spread alterations in gene-expression that promoted a survival like phenotype was observed in POR. The response in POR was accompanied with “costs of resistance” resulting in reduced growth efficiencies that are intrinsic to that group and were also observed during growth without antibiotic challenge, however, at lower levels. All strains showed similar minimal inhibitory concentrations and did not form phylogenetic groups, indicating that results cannot be attributed to distinct resistance levels or phylogenetic relationships, but are indeed based on the resistance mechanism. This issue also included a study from Ahvaz Jundishapur University of Medical Sciences of Iran. Saki et al. investigated the molecular epidemiology of carbapenem-resistant classic *K. pneumoniae* (CR-cKp) and carbapenemresistant hypervirulent *K. pneumoniae* (CR-hvKp) isolates in southwestern Iran from 2019 to 2021.

New carbapenemase genotype or subtypes have been emerging and spreading to a variety of bacterial species. Prah et al. from the Tokyo Medical and Dental University of Japan reported the emergence of a high-risk *Klebsiella michiganensis* (KO_408) clone disseminating carbapenemase genes. Carbapenemase-producing organisms are major contributors to the extensive spread of carbapenem resistance. They recovered KO_408 from the respiratory culture of a 71-year-old patient diagnosed with pneumonia and found that KO_408 was ascribed to a novel sequence type, ST256, and harbored resistance genes conforming to its MDR phenotype. The blaNDM-5 gene was localized on the approximately 44.9 kb IncX3 plasmid, which was transferable in the conjugal assay. The acquisition of pKO_4-NDM-5 did not impose any fitness burden and showed high stability in the host cells. However, transformants with pKO_4-NDM-5 were outcompeted by their host cells and transconjugants with the IncX3-blaOXA-181 plasmid. The genetic environment of blaNDM-5 in pKO_4-NDM-5 has been previously described. This study highlights the emergence of a high-risk Klebsiella michiganensis clone harboring carbapenemase genes and affirms that the recent spread of IncX3-blaNDM-5 plasmids might be due to their low fitness cost and stability but not their competitive prowess.

In Hangzhou, a Southern city of China, Wang et al. of the Second Affiliated Hospital of Zhejiang University School of Medicine reported the *Identification of a novel ceftazidime-avibactam-resistant KPC-2 Variant, KPC-123, in Citrobacter koseri following ceftazidime-avibactam treatment*. This novel KPC-123 consisted of 302 amino acids which differs from KPC-2 by two insertions after positions 179 (ins179_TY) and 270 (ins270_DDKHSEA), respectively. Whole-genome sequencing and genomic analysis revealed that blaKPC−123 within the “ISKpn27-blaKPC-ISKpn6” structure was located on a 93,814-bp conjugative plasmid that was almost identical to a blaKPC−2-carrying plasmid harbored in a *K. pneumoniae* isolate from the same sampling site of the patient, suggesting the transfer and *in vivo* evolution of this blaKPC-carrying plasmid. Carbapenem-resistant *K. pneumoniae* (CRKP) seriously threaten the efficacy of modern medicine with a high associated mortality rate and unprecedented transmission rate. Again in Hangzhou, Zhao et al. from Zhejiang Chinese Medical University reported the emergence of a NDM-1-producing ST25 *K. pneumoniae* strain causing neonatal sepsis. In another study, Guo et al. from the Center of Laboratory Medicine, First Affiliated Hospital of Chinese PLA General Hospital of China performed a genomic analysis of KPC-2-producing *K. pneumoniae* ST11 isolates at the Respiratory Department of the military tertiary hospital. From 2012 to 2018, the detection rate of the blaKPC-2-carrying CRKP raised rapidly from 3.3 to 28.1%. The KPC-2 producing *K. pneumoniae* ST11 were highly heterogeneous in the hospital. Transmission of these isolates was mainly mediated by twelve high-risk clones. The blaKPC-2-carrying plasmids and genetic environment of blaKPC-2 genes exhibited active evolution in *K. pneumoniae* ST11.

There are limited reports on the prevalence of carbapenemase-producing *Enterobacterales* (CPE) in aquatic environments and its association with clinical isolates. The study from Sung et al. team in the Busan Institute of Health and Environment of the Republic of Korea aimed to investigate the prevalence of CPE in a stream environment and its genetic relationship with clinical isolates in Korea. Among the total of 133 CRE strains isolated from the streams, *K. pneumoniae* was the most common CRE (45.9%), followed by *E. cloacae* complex (29.3%), *E. coli* (13.5%), *Raoultella ornithinolytica* (5.3%), and *C. freundii* (2.3%). Ninety (67.7%) isolates carried carbapenemase genes. KPC-2 (36.7%) and NDT-β-lactamase-5 (32.2%) were the common carbapenemases detected. The findings indicated that CPE was highly prevalent in streams and closely related to the isolates obtained from patients in South Korea. Investigators from the Gansu Provincial People's Hospital in Xining and Huashan Hospital in Shanghai of China investigated the molecular epidemiology and mechanism underlying ertapenem resistance of *K. pneumoniae* strains that are sensitive to meropenem and imipenem. Among the total 697 *K. pneumoniae* isolates, 7.4% of the tested CRKP strains were resistant only to ertapenem with no carbapenemase genes identified. Further exploration revealed that ertapenem resistance is likely related to ramR mutation. The function of ramR was confirmed using gene complementation to the original strain to determine the mechanism underlying ertapenem resistance of *K. pneumoniae* strains. Hence, active surveillance of carbapenem resistance and the underlying mechanisms, which may facilitate the prevention and control of the dissemination of resistance, is constantly needed.

## Author contributions

The author confirms being the sole contributor of this work and has approved it for publication.

## Dedication

This Research Topic is dedicated to Dr. Charles W. Stratton, a professor of Department of Pathology, Microbiology, and Immunology at the Vanderbilt University Medical Center in Nashville, Tennessee, the United States of America, who contributed as a co-associate editor and passed away during its preparation.

